# Giant panda seasonal adaptations in feeding strategies and blood physiology

**DOI:** 10.3389/fvets.2025.1703367

**Published:** 2025-12-08

**Authors:** Jiang Gu, Xiang Yu, Dunwu Qi, Rong Hou, Long Zhang, Guanwei Lan, Feifei Feng, Wenlei Bi, Fei Xue, Jiabin Liu, Chong Huang, Zusheng Li, Yanshan Zhou, Chao Chen, Wei Wu, Ping Li, Xi Yang, Mei Zhang, Hui He, Hong Yang, Rui Ma

**Affiliations:** 1 The Conservation of Endangered Wildlife Key Laboratory of Sichuan Province, Chengdu Research Base of Giant Panda Breeding, Chengdu, Sichuan, China; 2 Administration of Daxiangling Nature Reserve, Yaan, Sichuan, China

**Keywords:** giant panda, foraging strategy, blood physiology and biochemistry, adaptability, reintroduction

## Abstract

Understanding physiological adaptations of endangered giant pandas to seasonal changes is critical for improving conservation efforts, yet integrated analyses of blood parameters and feeding strategies remain limited. To decode seasonal adaptation mechanisms, we analyzed 36 monthly blood samples from 3 female pre-released training pandas alongside bamboo components across winter–spring and summer-autumn. Results showed significantly higher lymphocyte counts and urea levels, but lower serum aspartate aminotransferase, serum alkaline phosphatase, glucose and lipase in summer-autumn. Correspondingly, pandas consumed significantly more hemicellulose, crude ash, crude protein and minerals (e.g., potassium, calcium, magnesium, phosphorus, manganese, chromium, copper and zinc) in summer and autumn, while greater intake of starch, lignin, cadmium in winter–spring, reflecting a shift from stems to leaves. Key blood parameters correlated with bamboo component intake. These findings indicate a nutrient-driven strategy favoring anabolic metabolism in resource-rich seasons, providing physiological thresholds for improved conservation and release programs.

## Introduction

The giant panda, as a flagship species for global biodiversity conservation, human activities during the last glacial period of the Quaternary period and the Holocene epoch have caused the giant panda population to collapse severely, leading to its endangered status (1). To date, with the establishment of nature reserves, both the population of wild giant pandas and the area of their habitats in China have shown significant growth (2). Based on the increase in wild giant panda populations, the species has transitioned from endangered to vulnerable status. However, conservation efforts continue to face threats from various diseases, as well as challenges such as small population isolation and extinction risks (3–7). Reintroducing captive giant pandas into the wild is one of the key methods for revitalizing isolated small populations (8). However, the success rate of releasing captive pandas into the wild remains low (8). This is primarily due to their poor adaptability to natural environments and the lack of established, effective long-term monitoring systems (8–14). Research has shown that captive animals have become heavily dependent on favorable captive environments, resulting in low adaptability to wild conditions (15). Research on the adaptability of giant pandas has shown that their maintenance of low energy expenditure is closely associated with characteristics such as reduced activity levels and decreased thyroid hormone levels (16). The giant panda’s diet has become specialized to bamboo as an adaptation to its environment. It seasonally selects specific parts of bamboo, primarily based on nutritional and energy requirements, thereby maximizing the acquisition of sufficient nutrients and energy from its food (17, 18). Therefore, we believe that adaptive assessment holds significant importance for the reintroduction of captive giant pandas into the wild.

Artificial-assisted reintroduction is an effective method for increasing the size and genetic diversity of isolated small populations. This method has been used for American black bears (*Ursus thibetanus*) in India, sun bears (*Helarctos malayanus*) in Indonesia, American black bears in New Hampshire (19–24). Based on this method, existing research has demonstrated that after a three-month adaptation period following release, the gut microbiota transformation in giant pandas provides them with more diversified energy acquisition strategies better suited to the wild environment (8). Bi et al. (25) discovered that giant pandas exhibit elevated levels of hemoglobin and hematocrit in their blood as an adaptation to higher environmental altitudes. However, there is currently limited research on the relationship between physiological changes and adaptive responses in giant pandas.

Changes in blood physiological and biochemical indicators are commonly used to assess the health status of animals (26). For healthy animals, their environmental adaptability can be measured by their blood physiological and biochemical levels (25, 27, 28). Relevant studies indicate that blood parameters in giant pandas are influenced by factors such as age, sex, dietary intake, and season (29–32). Research has already been conducted on other wildlife. Whiteman et al. (33) found that polar bears (*Ursus maritimus*) inhabiting terrestrial habitats exhibited higher total white blood cell counts, neutrophil counts, and monocyte counts compared to ice-dwelling polar bears. No significant differences were observed in lymphocyte, eosinophil, basophil, or globulin levels, indicating a higher risk of infection (33). Sergiel et al. (34) found that brown bears (*Ursus arctos*) exhibit reduced urea levels and increased creatinine levels during winter, suggesting these serve as physiological indicators of hibernation. Randi et al. (35) found that serum enzyme levels such as aspartate aminotransferase and alanine aminotransferase decreased in brown bears (*Ursus arctos*) during hibernation, while triglycerides, cholesterol, and lipase levels increased, reflecting a lipid metabolism pattern during hibernation. However, there is currently no research on the seasonal adaptation of blood indicators in wild giant pandas.

We hypothesize that giant pandas have the ability to adapt to seasonal changes in the environment and climate. The aim of this study is to explore the adaptability of giant pandas to seasonal environmental changes from the perspectives of blood physiological and biochemical levels and feeding strategies. To verify this ability, we focused on pre-released training giant pandas, measuring blood physiological and biochemical indicators and the content of bamboo stem and leaf components consumed during different seasons, and conducting relevant statistical analyses. This research seeks to provide physiological threshold data for optimizing conservation and reintroduction programs.

## Materials and methods

### Subjects

The subjects of this study were three female adult pre-release training giant pandas (7 years old, *n* = 2; 9 years old, *n* = 1), all in good health with no illnesses throughout the year. All three pre-release training giant pandas lived within the adaptation area of the Daxiangling Nature Reserve, where their feeding, drinking, resting, and activities all took place in a natural wild environment.

### Study site

**Figure 1:**
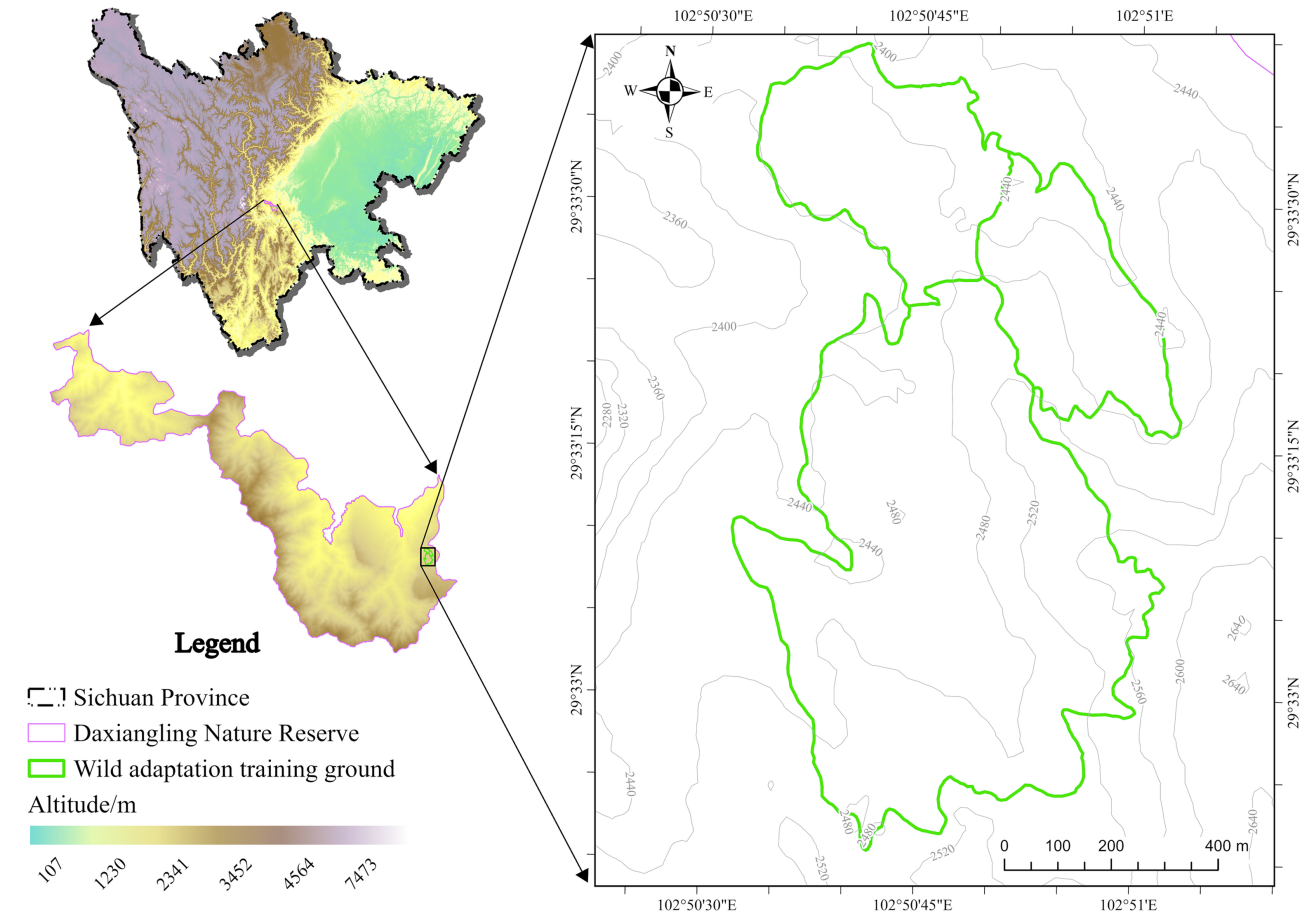
Site map of the wild adaptation area.

Three pre-release training giant pandas lived in the wild adaptation area, feeding on naturally growing Arundinaria faberi Rendle. The wild adaptation area is located within the Daxiangling Nature Reserve in the western Sichuan Basin, at an altitude of 2,456–2,495 meters, covering an area of 13.3 to 49.4 hectares, with an average annual temperature of 16 °C and an average relative humidity of 76% (Figure 1).

### Sample collection

In this study, all samples were collected from December 2021 to November 2022. The sample collection primarily involved the gathering of overnight fecal samples and bamboo samples for food nutritional component surveys. Owing to the successful implementation of artificial assisted soft-release training (Invention Patent in China: A domestication method of wild release and artificial intervention of giant panda; patent number: ZL 2017 10576548.4), researchers maintained close contact with the giant pandas over extended periods (8). This approach facilitated the immediate collection of fecal samples following defecation. Additionally, under this patent, we utilized Beidou satellite collars (HQAI-M, Global Messenger Technology Co., Ltd., Hubei, China) to collect hourly activity locations of the pandas and track their movements to identify previous activity areas. In this study, collection personnel strictly adhered to the sample collection protocols, wearing disposable surgical masks and polyethylene (PE) gloves.

Utilizing location data from satellite collars fitted on giant pandas, we conducted monthly surveys of their foraging areas, focusing on the areas visited the previous day. Based on feeding traces and patterns, we collected 100 g each of arrow bamboo leaves and culms from the surrounding area, cleaning and placing them in PE tubes. These samples were transported to the laboratory with ice packs and stored at −80 °C in an ultra-low temperature freezer until further nutritional component analysis. As there were no observed instances of the study subjects consuming shoots throughout the year, this study did not involve the collection or compositional analysis of bamboo shoot samples. In addition, on the 5th, 15th, and 25th of each month, we surveyed the pandas’ resting areas from the previous day. We searched for fecal piles containing more than 20 units feces, which are indicative of the pandas’ comprehensive feeding strategy on different parts of the bamboo. These fecal piles were collected entirely into PE bags and transported to the laboratory at ambient temperature for storage (Supplementary Table S1).

Based on the feeding strategies of giant pandas, the seasons are divided into two groups: winter–spring season (December, January–May) and summer-autumn season (June–November).

Blood samples are collected once a month during physical examinations using a non-anesthetic blood collection method. Each giant panda individual was well-trained for blood collection and completed the procedure without anesthesia. Blood collection was conducted using disposable sterile needles for venipuncture. Each panda contributed two types of blood samples, 1 mL blood sample was placed in an EDTA tube for hematological analysis, another 1 mL in a heparin sodium anticoagulant tube for biochemical analysis. Blood samples were centrifuged at 3,000 rpm for 10 min and stored at 4 °C and immediately sent for testing and analysis.

### Blood physiological and biochemical indicator measurement

The blood samples are then transported to the Blood Testing Center of the People’s Hospital of Yingjing County, Ya’an City, Sichuan Province, within 6 h for routine blood tests and biochemical analysis (10). Blood physiological indicator measurements mainly include white blood cells (WBC), neutrophils (NEU), monocytes (MON), lymphocytes (LYM#), red blood cells (RBC), hemoglobin (HGB), red blood cell hematocrit (HCT), platelets (PLT), and platelet hematocrit (PCT), totaling 9 parameters. Complete hematological analysis was done using the ProCyte Dx automated hematology analyzer (IDEXX Laboratories, Westbrook, ME, United States). Blood biochemical parameter measurements primarily include serum alanine aminotransferase (ALT), serum aspartate aminotransferase (AST), serum alkaline phosphatase (ALP), serum gamma-glutamyl transpeptidase (GGT), serum total protein (TP), serum albumin (ALB), serum globulin (GLOB), albumin-to-globulin ratio (A/G), serum urea (UREA), creatinine (CREA), uric acid (UA), blood glucose (GLU), serum triglycerides (TG), serum total cholesterol (TCHOL), serum lactate dehydrogenase (LDH), serum amylase AMY, serum lipase (LPS), potassium (K), natrium (Na), chlorine (Cl), calcium (Ca), magnesium (Mg), and phosphorus (P), totaling 23 parameters. Blood biochemical parameters were measured using the FUJI automatic dry chemistry analyzer (DRI-CHEM NX500iVC, FUJIFILM, Tokyo, Japan).

### Analysis of the feeding strategy of giant pandas

All the fecal piles samples collected from each individual were manually broken down and spread evenly on ceramic plates. These samples were then placed in an oven and heated at 65 °C for 72 h to remove moisture. Following this, the bamboo leaf and culm residues within the feces were separated manually and weighed individually (36). The monthly intake ratio of bamboo leaves to culms was subsequently calculated based on these measurements (37, 38).

The Bamboo culm and leaf samples were sent to Baihui Organisms Technology Co., Ltd. (Sichuan, China) for nutritional composition analysis. The determination of nutritional components followed established methods from previous studies. Specifically, the methods described by Knott et al. (39) were employed to measure crude fat, starch, crude ash, moisture, crude protein content, tannin, and total phenol. Soluble sugar content was determined following the method outlined by Van Soest et al. (40). The measurement of cellulose, lignin, and hemicellulose was conducted according to the methodology of Kehoe et al. (32). Eight trace elements, including zinc (Zn), copper (Cu), cadmium (Cd), chromium (Cr), lead (Pb), cobalt (Co), molybdenum (Mo), and Titanium (Ti) were analyzed by the Inductively Coupled Plasma Mass Spectrometry (ICP-MS), following the approach detailed in the study by Wei et al. (41). Seven macro elements, including potassium (K), natrium (Na), calcium (Ca), magnesium (Mg), phosphorus (P), ferrum (Fe), and manganese (Mn), was performed using the Inductively Coupled Plasma Atomic Emission Spectroscopy (ICP-AES) according to the methodology described in the study by Wang et al. (42). Calculate the intake of bamboo components based on the measured content of bamboo stems and leaves and their seasonal consumption proportions (Supplementary Table S2).

### Statistical analysis

The collected data were analyzed using SPSS 27.0 statistical software. All results are expressed as mean ± standard deviation (Mean ± SD). Linear mixed models (LMM) were used for statistical analysis of blood parameters, with season as a fixed blocking factor and panda ID as a random intercept to account for repeated measures across individuals. Fixed-effect tests with 0.01 < *p* < 0.05 indicated significant differences, and *p* < 0.01 indicated extremely significant differences. Random-effect variance with *p* > 0.05 indicated no heterogeneity between groups (43). Independent samples Kruskal–Wallis tests for statistical analysis of bamboo component intake, followed by Dunn’s *post hoc* test for pairwise comparisons, and applied the false discovery rate (FDR) correction to adjust the *p*-values obtained from Dunn’s post hoc test. With 0.01 < *p*_FDR_ < 0.05 indicating statistically significant differences and *p*_FDR_ < 0.01 indicating extremely significant differences. The Spearman’s correlation coefficient was used for statistical analysis of the correlation between blood indicators and bamboo component intake. The correlation threshold was 0.30 (44). A positive correlation coefficient *R* indicates a positive correlation, while a negative correlation coefficient *R* indicates a negative correlation. 0.01 < *p* < 0.05 indicates a significant correlation, and *p* < 0.01 indicates a highly significant correlation. A multiple linear regression model was employed to analyze the combined effects of bamboo nutrients on blood indicator levels, identifying the primary nutrients with the most significant influence. These correlations were corrected for multiple testing using the R (v3.6.1) package psych and visualized as heatmaps in GraphPad Prism 9.

## Result

### Analysis of seasonal differences in the physiological and biochemical levels of giant panda blood

Seasonal changes have a significant impact on the physiological indicators of giant pandas’ blood (Table 1). LYM# levels are higher in summer-autumn seasons than in winter–spring seasons, the fixed-effect test shows 0.01 < *p* < 0.05. Seasonal changes significantly affect blood biochemical indicators (Table 1), serum AST and ALP are extremely significantly lower in summer-autumn seasons than in winter–spring seasons, with fixed-effect tests yielding *p* < 0.01. UREA levels were significantly higher in summer-autumn seasons than in winter–spring seasons, with a fixed-effects test *p* < 0.01. GLU and LPS levels were significantly lower in summer and autumn seasons than in winter–spring seasons, with a fixed-effects test 0.01 < *p* < 0.05. Among them, all random effect variances *p* > 0.05, indicating no heterogeneity between groups.

**Table 1:** Analysis of differences in blood physiological and biochemical indicators between giant pandas in winter–spring seasons and in summer-autumn seasons.

Indicator	Unit	Winter–spring seasons (WS)		Summer-autumn seasons (SA)		Fixed-effect tests’ *p* between
		Mean	SD	Mean	SD	WS and SA
WBC	10^9^/L	7.235	1.074	7.591	1.036	0.281
NEU	10^9^/L	4.737	0.810	4.182	1.361	0.146
LYM#	10^9^/L	1.843	0.536	2.491	1.117	0.019^*^
MON	10^9^/L	0.376	0.172	0.438	0.227	0.270
RBC	10^12^/L	6.831	0.722	6.501	0.589	0.142
HGB	g/L	136.611	14.055	129.278	12.732	0.110
HCT	%	0.387	0.041	0.369	0.034	0.166
PLT	10^9^/L	453.000	137.179	506.167	156.726	0.243
PCT	%	0.247	0.090	0.298	0.100	0.078
ALT	U/L	107.028	35.282	94.756	17.559	0.165
AST	U/L	109.839	43.150	72.861	21.172	0.002^**^
ALP	U/L	176.111	70.712	144.222	41.716	0.009^**^
GGT	U/L	9.022	5.323	7.433	1.405	0.229
TP	g/L	67.268	5.330	68.285	3.314	0.461
ALB	g/L	34.611	2.806	34.439	2.709	0.803
GLOB	g/L	32.657	3.665	34.459	3.857	0.142
A/G	–	1.068	0.128	1.029	0.161	0.363
UREA	μmol/L	7.037	2.885	9.633	3.623	0.007^**^
CREA	μmol/L	143.678	26.666	164.244	38.279	0.067
UA	μmol/L	31.817	13.860	38.450	15.839	0.190
GLU	μmol/L	3.974	1.564	2.952	1.557	0.048^*^
TG	μmol/L	1.766	0.759	1.853	1.076	0.778
TCHOL	μmol/L	5.486	1.108	5.267	1.396	0.461
LDH	U/L	817.858	221.637	770.628	214.625	0.289
AMY	U/L	895.361	223.931	972.356	178.195	0.219
LPS	U/L	8.356	2.242	6.972	1.302	0.020^*^
K	mmol/L	5.120	1.135	4.823	0.671	0.329
Na	mmol/L	126.267	3.406	125.094	2.126	0.213
CL	mmol/L	97.461	2.065	97.461	2.143	1.000
Ca	mmol/L	2.091	0.159	2.095	0.125	0.923
Mg	mmol/L	1.100	0.105	1.155	0.096	0.101
P	mmol/L	1.295	0.346	1.298	0.239	0.977

Linear mixed models (LMM) were used for statistical analysis of blood parameters. ^*^Indicates that the fixed effect test 0.01 < *p* < 0.05, the difference is significant; ^**^indicates that the fixed effect test *p* < 0.01, the difference is extremely significant. Among them, all random effect variances *p* > 0.05, indicating no heterogeneity between groups.

### Analysis of the feeding strategy of giant pandas

**Figure 2:**
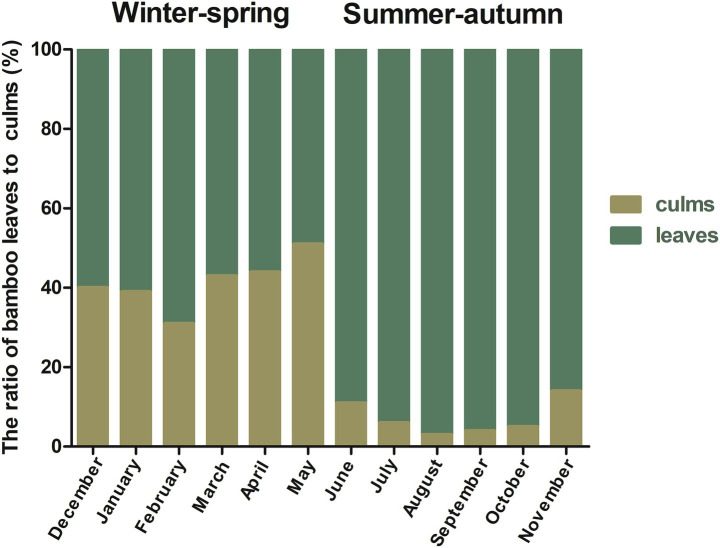
Proportion of bamboo culms and leaves consumed by release giant pandas.

Seasonal changes affect the feeding strategies of giant pandas. In winter–spring, they feed on a mixture of bamboo culms and leaves, while in summer-autumn, they mainly feed on bamboo leaves (Figure 2), which in turn affects their intake of bamboo nutrients and Statistical analysis revealed seasonal variations in the intake of certain nutrients (Table 2). Starch content (*p*_FDR_ = 0.031) intake was significantly lower in summer-autumn seasons than in winter–spring seasons. Lignin content (*p*_FDR_ < 0.01) and Cd intake (*p*_FDR_ = 0.002) were extremely significantly lower in summer-autumn seasons than in winter–spring seasons. In summer-autumn seasons, the intake of hemicellulose content (*p*_FDR_ = 0.004), crude ash content (*p*_FDR_ < 0.01), crude protein content (*p*_FDR_ < 0.01), Ca (*p*_FDR_ < 0.01), K (*p*_FDR_ < 0.01), Mg (*p*_FDR_ < 0.01), Mn (*p*_FDR_ = 0.001), P (*p*_FDR_ < 0.01), Cr (*p*_FDR_ = 0.002), Cu (*p*_FDR_ < 0.01), and Zn (*p*_FDR_ = 0.004) intake were extremely significantly higher in summer-autumn seasons than in winter–spring seasons.

**Table 2:** Analysis of differences in the intake of substances in bamboo consumed by giant pandas during the winter–spring seasons and the summer-autumn seasons.

Food compounds	Winter–spring seasons (WS)		Summer-autumn seasons (SA)		Adjusted *p*-value (FDR)
	Mean	SD	Mean	SD	WS and SA
Crude fat content (%)	0.043	0.009	0.039	0.006	0.164
Soluble sugar content (%)	0.056	0.013	0.049	0.014	0.635
Starch content (%)	0.094	0.020	0.078	0.013	0.031^*^
Cellulose content (%)	0.212	0.022	0.224	0.017	0.082
Lignin content (%)	0.128	0.009	0.103	0.015	<0.001^***^
Hemicellulose content (%)	0.320	0.015	0.339	0.025	0.004^**^
Crude ash content (%)	0.024	0.013	0.043	0.011	<0.001^***^
Moisture content	0.091	0.013	0.091	0.014	0.924
Crude protein content (%)	0.097	0.011	0.144	0.024	<0.001^***^
Cyanide (mg/Kg)	0.184	0.086	0.175	0.144	0.195
Ca (mg/Kg)	1924.375	233.787	2766.471	423.233	<0.001^***^
Fe (mg/Kg)	564.130	250.597	471.800	93.753	0.164
K (mg/Kg)	4846.573	708.124	6781.160	1185.830	<0.001^***^
Mg (mg/Kg)	676.157	84.505	912.114	117.371	<0.001^***^
Mn (mg/Kg)	381.926	97.562	510.652	96.561	0.001^**^
Na (mg/Kg)	108.256	25.313	110.669	35.448	1.000
P (mg/Kg)	1080.655	117.795	1730.091	173.994	<0.001^***^
Cd (mg/Kg)	0.078	0.022	0.054	0.024	0.002^**^
Cr (mg/Kg)	16.344	4.984	21.999	5.523	0.002^**^
Cu (mg/Kg)	11.905	3.561	17.101	3.808	<0.001^***^
Pb (mg/Kg)	3.905	1.310	3.861	1.383	0.800
Zn (mg/Kg)	54.000	8.627	64.295	9.878	0.004^**^
Co2286 (mg/Kg)	0.353	0.136	0.399	0.110	0.206
Co2388 (mg/Kg)	0.773	0.172	0.761	0.222	0.752
Mo2020 (mg/Kg)	0.197	0.045	0.235	0.070	0.137
Mo2816 (mg/Kg)	1.641	0.409	1.839	0.397	0.242
Ti3234 (mg/Kg)	19.720	10.554	23.105	9.781	0.282
Ti3349 (mg/Kg)	20.757	11.051	19.674	6.809	0.800
Tannin (mmol/Kg)	6.337	1.507	6.678	1.387	0.296
Total phenol (mmol/Kg)	13.891	1.694	14.055	2.194	0.613

^*^Indicates 0.01 < p_FDR_ < 0.05; ^**^indicates 0.001 < p_FDR_ < 0.01; ^***^indicates p_FDR_ < 0.001.

### Correlation analysis between bamboo intake and blood indicators in giant pandas

**Figure 3:**
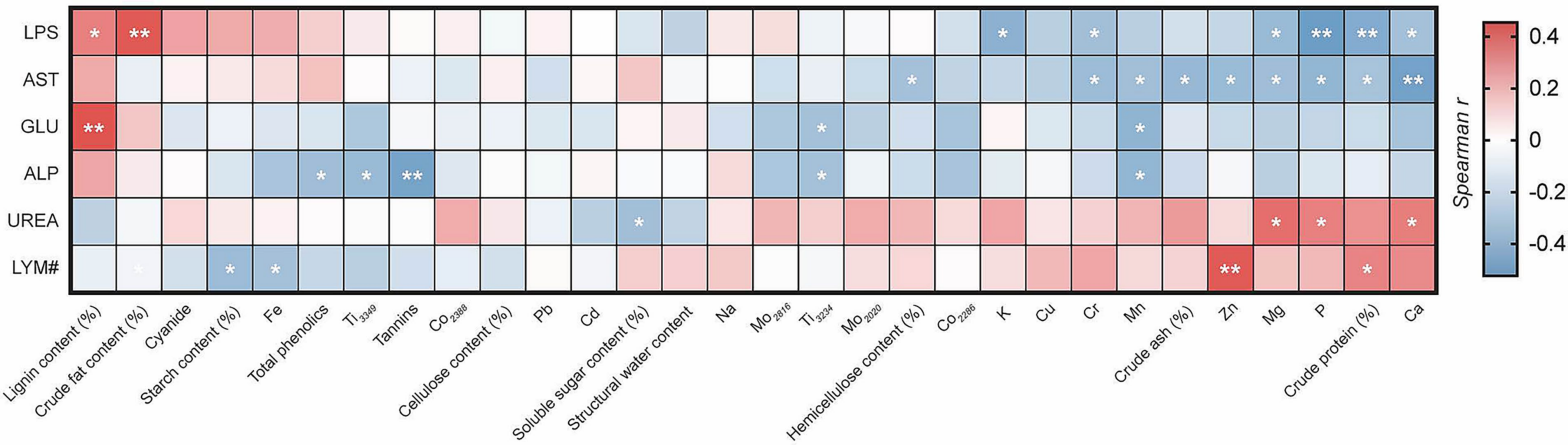
Correlation analysis heatmap. The images only display blood markers significantly correlated with nutritional components. Red indicates a positive correlation, blue indicates a negative correlation, ^*^indicates significant, and ^**^indicates extremely significant.

Correlation analysis found that the intake of bamboo substances was correlated with certain physiological and biochemical levels in the blood of giant pandas (Figure 3). There was a significant positive correlation between LYM# and crude protein content intake (*R* = 0.333, *p* < 0.05), and a highly significant positive correlation between LYM# and Zn intake (*R* = 0.439, *p* < 0.01). There was a significant negative correlation between LYM# and starch content (*R* = −0.366, *p* < 0.05), Fe intake (*R* = −0.332, *p* < 0.05). AST was significantly negatively correlated with hemicellulose content (*R* = −0.334, *p* < 0.05), crude ash content (*R* = −0.386, *p* < 0.05), crude protein content (*R* = −0.330, *p* < 0.05), Mg (*R* = −0.350, *p* < 0.05), Mn (*R* = −0.348, *p* < 0.05), P (*R* = −0.398, *p* < 0.05), Cr (*R* = −0.364, *p* < 0.05), and Zn intake (*R* = −0.369, *p* < 0.05), and extremely significant negative correlations with Ca intake (*R* = −0.505, *p* < 0.01); ALP levels showed significant negative correlations with Mn (*R* = −0.397, *p* < 0.05), Ti3234 (*R* = −0.335, *p* < 0.05), Ti3349 (*R* = −0.375, *p* < 0.05), and total phenolic intake (*R* = −0.352, *p* < 0.05), and a highly significant negative correlation with tannin intake (*R* = −0.494, *p* < 0.01). UREA showed a significant negative correlation with soluble sugar content intake (*R* = −0.345, *p* < 0.05), and a significant positive correlation with Ca (*R* = 0.346, *p* < 0.05), Mg (*R* = 0.388, *p* < 0.05), P intake (*R* = 0.341, *p* < 0.05). GLU showed a highly significant positive correlation with lignin content intake (*R* = 0.454, *p* < 0.01) and a significant negative correlation with Mn (*R* = −0.410, *p* < 0.05), Ti3234 intake (*R* = −0.332, *p* < 0.05). LPS showed a highly significant positive correlation with crude fat content intake (*R* = 0.442, *p* < 0.01), a significant positive correlation with lignin content intake (*R* = 0.345, *p* < 0.05), and extremely significant negative correlations with crude protein content (*R* = −0.448, *p* < 0.01), P intake (*R* = −0.524, *p* < 0.01), and significant negative correlations with Ca (*R* = −0.336, *p* < 0.05), K (*R* = −0.419, *p* < 0.05), Mg (*R* = −0.372, *p* < 0.05), and Cr2835 intake (*R* = −0.346, *p* < 0.05).

Multivariate linear regression analysis was conducted using bamboo nutrient intake as independent variables and blood indicator levels as dependent variables to identify the primary nutrients exerting the most significant influence (Table 3). Results revealed: dietary Zn intake showed a significant positive correlation with LYM# levels (*p* = 0.027), Ca intake exhibited a highly significant negative correlation with AST levels (*p* = 0.001), tannin intake showed a highly significant negative correlation with ALP (*p* = 0.002), soluble sugar content intake correlated significantly negatively with UREA (*p* = 0.029), lignin content intake correlated highly significantly positively with GLU (*p* = 0.005), and crude fat content intake correlated highly significantly positively with LPS (*p* < 0.001).

**Table 3:** Multivariate linear regression results of blood marker levels in giant pandas and dietary nutrient intake.

Blood marker levels vs. bamboo nutrient intake	*R*-squared	*B*	Beta	*t*	*p*	VIF
LYM# vs. Zn	0.136	0.032	0.368	2.309	0.027^*^	1.000
AST vs. Ca	0.266	−0.036	−0.516	−3.511	0.001^**^	1.000
ALP vs. Tannin	0.247	−20.535	−0.497	−3.337	0.002^**^	1.000
UREA vs. Soluble sugar content	0.133	−93.806	−0.364	−2.282	0.029^*^	1.000
GLU vs. Lignin content	0.212	42.764	0.460	3.024	0.005^**^	1.000
LPS vs. Crude fat content	0.328	141.600	0.573	4.074	<0.001^***^	1.000

^*^Indicates 0.01 < *p* < 0.05; ^**^indicates 0.001 < *p* < 0.01; ^***^indicates *p* < 0.001.

## Discussion

Blood physiology and biochemical levels reflect the long-term adaptation of animals to their living environment, reflecting not only their growth and development but also their health status. Normal blood physiological and biochemical levels are relatively stable, and normal fluctuations in these indicators indicate metabolic and physiological changes in the animal’s body. Our study analyzed seasonal variations in physiological and biochemical indicators in the blood of pre-release training giant pandas. Results revealed that during summer-autumn, levels of LYM and CRUA significantly increased, while levels of AST, ALP, GLU, and LPS decreased.

Blood lymphocytes, as immune cells responsible for the animal body’s defense against pathogens, have their resource supply directly influenced by seasonal energy and nutrient intake strategies (45). Our study found that LYM# levels of pre-release training giant pandas were higher during summer and autumn than during winter and spring, which is similar to the findings from Tsessarskaia and Burkovskaya (46). In their study of outdoor-reared dogs, who observed higher LYM# levels in these dogs from May to September compared to November to March of the following year. Similar to findings from studies on terrestrial-phase polar bears by Whiteman et al. (33), their research revealed that LYM# levels in terrestrial-phase polar bears were higher than during the ice-phase, indicating increased pathogen infection risk during the terrestrial phase. In contrast to the findings of Linhua et al. (47) on captive giant pandas, Linhua et al. discovered that age had a highly significant effect on LYM# levels in captive giant pandas, while seasonality showed no significant difference. Therefore, we speculate that the LYM# levels are associated with adapting to seasonal environmental changes and the high risk of pathogen infection during summer-autumn (48–50). Our study also analyzed the correlation between the intake of bamboo constituents and blood indicators, revealing that crude protein and zinc intake during summer-autumn were higher than in winter–spring. These were positively correlated with LYM# levels and negatively correlated with starch intake. Crude protein is a direct source of amino acids required for LYM# proliferation, and Zn is an essential mineral element for lymphocyte differentiation. Increasing the crude protein and Zn content of food provides the material basis for LYM# production in the body (51–53). Starch, as the main source of energy for animal organisms, affects the metabolism of immune cells through blood sugar levels and insulin signaling. A high-starch diet may cause short-term fluctuations in blood sugar levels and inhibit LYM# activity (54). It also indicates that wild giant pandas primarily consume bamboo leaves during summer-autumn, increasing their intake of crude protein and zinc, thereby providing the material basis for LYM# production. In winter–spring, reduced pathogen infection pressure leads to mixed consumption of bamboo stems and leaves, with a relative increase in starch intake. This may suppress the activity of blood LYM#, reducing the maintenance of high lymphocyte counts, primarily to meet the body’s energy demands for adapting to low-temperature environments.

This study found that serum AST, serum ALP, serum LPS, and GLU levels in pre-release training giant pandas were lower during summer-autumn than during winter–spring, while UREA levels were higher during summer-autumn than during winter–spring. AST is a key enzyme in the crosstalk between protein and carbohydrate metabolism, transferring amino groups from amino acids into gluconeogenesis and the citric acid cycle via transamination reactions (55, 56). Our findings contrast with those of Randi et al. (35) in brown bears, they observed decreased serum enzyme levels such as aspartate aminotransferase (AST) and elevated LPS levels in hibernating brown bears. This reflects reduced metabolic function during hibernation, with metabolic patterns shifting toward lipid metabolism. ALP is a class of phospholipase enzymes widely distributed throughout the body’s tissues, catalyzing the hydrolysis of organic phospholipids. Zinc and magnesium serve as essential cofactors for their activity (57, 58). LPS is a key enzyme that breaks down fats and lipids into fatty acids and glycerol, aiding in the maintenance of normal gallbladder function. Its serum levels reflect the intensity of the body’s lipolytic metabolism (59, 60). This finding is consistent with the results of Jiang et al. (61). in captive giant pandas, who observed that LPS levels in captive giant pandas were higher in spring than in other seasons, while ALP levels were lower in autumn than in other seasons. GLU is the body’s most direct source of energy, and its levels are regulated by the dynamic balance of “intake-consumption-storage” (62). Similar to the findings of Yunxiao et al. (63) in cynomolgus monkeys (*Macaca mulatta*), they observed that blood GLU levels in cynomolgus monkeys exhibited a cyclical fluctuation pattern, with the lowest values occurring in summer, the highest in winter, and intermediate levels in spring and autumn. The production of UREA directly depends on the breakdown of proteins or amino acids. The level of urea in the blood mainly reflects the intensity of protein metabolism and excretion efficiency (64). Similar to findings from Sergiel et al. (34) in brown bears, they observed reduced UREA levels and increased CREA levels in wintering brown bears, suggesting these serve as physiological indicators of hibernation in brown bears. It is speculated that the reason may be that brown bears have a low basal metabolic rate during hibernation, whereas giant pandas do not hibernate. During the winter–spring period, low temperatures and food scarcity drive the body to predominantly adopt catabolic metabolism. Specifically, the elevation of lipase (LPS) levels facilitates the breakdown of stored fat, while increased glucose (GLU) levels guarantee energy supply—effectively meeting the body’s demands for thermogenesis and survival (65–67). During this phase, metabolic activity intensifies; concurrently, enhanced cellular permeability leads to the release of aspartate aminotransferase (AST) and alkaline phosphatase (ALP) into the bloodstream, resulting in relatively higher serum AST and ALP levels compared to those observed in summer and autumn (68). In contrast, the warm, mild summer and autumn seasons are characterized by abundant food availability. During this period, metabolic rates maintain basal levels, thereby imposing minimal metabolic stress on tissue cells. The body then shifts its focus to energy storage and growth: abundant dietary protein is not only broken down to generate energy but also utilized for synthesizing fat reserves, ultimately leading to relatively elevated serum urea (UREA) levels (69, 70).

Based on the results of this study’s bamboo material composition intake analysis, the intake of hemicellulose, crude ash, crude protein, Ca, K, Mg, Mn, P, Cr, Cu, and Zn was higher in summer-autumn than in winter–spring, while the intake of starch, lignin, and Cd was lower in summer-autumn than in winter–spring, Noting that summer-autumn offer a richer variety of nutritious foods. The correlation analysis results of this study indicate that crude protein, Ca, Mg, and P all exhibit negative correlations with serum AST and LPS levels, while showing positive correlations with serum UREA levels. Crude protein from plants is broken down into ammonia by the animal body and converted into urea through the urea cycle. Magnesium (Mg) promotes urea production by activating key enzymes in this cycle (71), while phosphorus (P), as a core component of ATP, provides sufficient ATP for the urea cycle (72). This suggests that higher intake of crude protein, Mg, and P during summer and autumn maintains the body’s urea cycle, resulting in relatively elevated blood urea levels (73). Calcium, acting as an active cofactor for LPS, works with magnesium to maintain cellular homeostasis and antioxidant capacity, while interacting with phosphorus to preserve calcium-phosphorus equilibrium. This reduces mineral deposition and disruptions in lipid metabolism, thereby decreasing cellular release of AST and LPS (74). It also suggests that serum AST and LPS levels are relatively lower in summer and autumn compared to winter and spring, which correlates with the intake of animal nutrients. These nutrients synergistically provide the material foundation required for metabolism and maintain cell membrane integrity, thereby reducing the release of AST and LPS into the bloodstream (75).

The physiological adaptation of pre-release training giant pandas is crucial for successful reintroduction. Currently, their physiological adaptability is primarily reflected in the evolution of their gut microbiome and energy metabolism mechanisms, with few studies examining adaptation from a physiological perspective (76–78). This study investigated the adaptive responses of pre-release training giant pandas to seasonal environmental changes through blood physiological and biochemical parameters and foraging strategies. It revealed that during the wild-release training period, pandas adjusted their foraging strategies to accommodate seasonal variations in the wild environment, which in turn influenced their blood physiological and biochemical levels. These findings provide reference values for health monitoring. Additionally, further operational management protocols will be developed for habitat selection in reintroduction training grounds, monitoring of the reintroduction process, and evaluation of its effectiveness, ensuring that pre-release training giant pandas can adapt to seasonal changes in the wild.

This study has several limitations: (1) the small sample size (36 blood samples from 3 individuals) limits the statistical power and generalizability of our findings. Though the sample size aligns with common practices in endangered species research, small samples may amplify the influence of individual variability or outliers. Future studies should prioritize expanding the number of sampled individuals and ensuring balanced group sizes to validate our observations (8–10). (2) This study standardized the age groups, gender, diet, and living environments of the research subjects, thereby reducing the influence of multiple factors. However, further research is needed to explore the effects across different age groups and genders. (3) Our study suggests that seasonal variations in blood indicators correlate with dietary intake of specific substances. Nevertheless, comprehensive analysis incorporating environmental and meteorological factors remains necessary. In future research, we suggest: (1) while increasing the number of sample donors and distinguishing between gender and age groups, we will conduct in-depth investigations into habitat, climate, and activity intensity to further explore the physiological factors influencing giant pandas’ adaptation to wild environments. (2) Collect blood and fecal samples from study subjects for metabolomics and transcriptomics analysis, employing a multi-omics approach to uncover the mechanisms by which giant pandas adapt to seasonal environmental changes.

## Conclusion

This study found that pre-release training giant pandas have developed a nutrition-driven foraging strategy to adapt to seasonal changes in the wild: consuming both bamboo stems and leaves during winter–spring, and feeding primarily on bamboo leaves during summer-autumn. During summer-autumn, abundant food resources increase intake of nutrients such as hemicellulose, crude protein, Ca, Mg, P, and Zn. This strategy tends to promote anabolic metabolism, resulting in relatively elevated blood urea levels. During winter–spring when food resources are scarce, this strategy tends to promote catabolism, regulating elevated levels of blood AST, LPS, and GLU to ensure energy supply and cold resistance. These findings indicate a nutrient-driven strategy favoring anabolic metabolism in resource-rich seasons, providing physiological thresholds for improved conservation and release programs.

## Data Availability

The original contributions presented in the study are included in the article/Supplementary material, further inquiries can be directed to the corresponding author.
