# Supplementary material for: Giant panda seasonal adaptations in feeding strategies and blood physiology
Source: Front Vet Sci. 2025 Dec 8;12:1703367. doi: 10.3389/fvets.2025.1703367 (PMC12719280; doi:10.3389/fvets.2025.1703367)
Supplement: Supplementary file 1 [file Table_1.DOCX]

**Table S1.** Information on Collecting Fecal Samples from pre-release training giant pandas

| **Date** | **Name** | **Samples** | **Quantity (units)** |
| --- | --- | --- | --- |
| 2021.12 | PlQ | Feces | 67 |
|  | PlH |  | 66 |
|  | PlX |  | 71 |
| 2022.1 | PlQ | Feces | 69 |
|  | PlH |  | 67 |
|  | PlX |  | 66 |
| 2022.2 | PlQ | Feces | 62 |
|  | PlH |  | 73 |
|  | PlX |  | 61 |
| 2022.3 | PlQ | Feces | 67 |
|  | PlH |  | 64 |
|  | PlX |  | 65 |
| 2022.4 | PlQ | Feces | 64 |
|  | PlH |  | 76 |
|  | PlX |  | 72 |
| 2022.5 | PlQ | Feces | 68 |
|  | PlH |  | 61 |
|  | PlX |  | 65 |
| 2022.6 | PlQ | Feces | 68 |
|  | PlH |  | 81 |
|  | PlX |  | 70 |
| 2022.7 | PlQ | Feces | 68 |
|  | PlH |  | 69 |
|  | PlX |  | 65 |
| 2022.8 | PlQ | Feces | 73 |
|  | PlH |  | 74 |
|  | PlX |  | 68 |
| 2022.9 | PlQ | Feces | 71 |
|  | PlH |  | 62 |
|  | PlX |  | 76 |
| 2022.10 | PlQ | Feces | 65 |
|  | PlH |  | 77 |
|  | PlX |  | 65 |
| 2022.11 | PlQ | Feces | 69 |
|  | PlH |  | 61 |
|  | PlX |  | 69 |
