# Supplementary material for: Giant panda seasonal adaptations in feeding strategies and blood physiology
Source: Front Vet Sci. 2025 Dec 8;12:1703367. doi: 10.3389/fvets.2025.1703367 (PMC12719280; doi:10.3389/fvets.2025.1703367)
Supplement: Supplementary file 2 [file Table_2.DOCX]

**Table S2.** Detection results and intake of food compounds in bamboo culms and leaves in the WS and SA group

| **Foodcompounds** | **Culms inWS** | | **Culms inSA** | | **Leaves inWS** | | **Leaves in SA** | | **WS intake of Culms** | | **SA intake of Culms** | | **WS intake of Leaves** | | **SA intake of Leaves** | |
| --- | --- | --- | --- | --- | --- | --- | --- | --- | --- | --- | --- | --- | --- | --- | --- | --- |
|  | **Mean** | **SD** | **Mean** | **SD** | **Mean** | **SD** | **Mean** | **SD** | **Mean** | **SD** | **Mean** | **SD** | **Mean** | **SD** | **Mean** | **SD** |
| Crude fat content (%) | 0.033 | 0.013 | 0.030 | 0.010 | 0.050 | 0.010 | 0.040 | 0.006 | 0.014 | 0.008 | 0.002 | 0.001 | 0.029 | 0.004 | 0.037 | 0.005 |
| Soluble sugar content (%) | 0.048 | 0.015 | 0.031 | 0.005 | 0.062 | 0.011 | 0.050 | 0.016 | 0.019 | 0.006 | 0.002 | 0.002 | 0.037 | 0.011 | 0.047 | 0.015 |
| Starch content (%) | 0.124 | 0.035 | 0.100 | 0.031 | 0.071 | 0.007 | 0.076 | 0.012 | 0.052 | 0.022 | 0.008 | 0.006 | 0.041 | 0.007 | 0.071 | 0.011 |
| Cellulose content (%) | 0.216 | 0.044 | 0.248 | 0.043 | 0.212 | 0.033 | 0.221 | 0.018 | 0.088 | 0.024 | 0.019 | 0.013 | 0.124 | 0.024 | 0.205 | 0.020 |
| Lignin content (%) | 0.170 | 0.010 | 0.178 | 0.018 | 0.098 | 0.010 | 0.097 | 0.016 | 0.070 | 0.013 | 0.013 | 0.008 | 0.058 | 0.010 | 0.090 | 0.016 |
| Hemicellulose content (%) | 0.328 | 0.032 | 0.324 | 0.051 | 0.311 | 0.016 | 0.340 | 0.025 | 0.137 | 0.037 | 0.024 | 0.016 | 0.183 | 0.030 | 0.315 | 0.032 |
| Crude ash content (%) | 0.012 | 0.003 | 0.011 | 0.004 | 0.034 | 0.023 | 0.045 | 0.011 | 0.005 | 0.002 | 0.001 | 0.000 | 0.019 | 0.012 | 0.042 | 0.011 |
| Moisture content | 0.087 | 0.012 | 0.093 | 0.013 | 0.093 | 0.021 | 0.091 | 0.014 | 0.036 | 0.010 | 0.007 | 0.004 | 0.055 | 0.016 | 0.084 | 0.016 |
| Crude protein content (%) | 0.032 | 0.006 | 0.029 | 0.004 | 0.142 | 0.009 | 0.153 | 0.025 | 0.013 | 0.005 | 0.002 | 0.002 | 0.083 | 0.013 | 0.142 | 0.024 |
| Cyanide (mg/Kg) | 0.165 | 0.132 | 0.331 | 0.171 | 0.184 | 0.121 | 0.169 | 0.171 | 0.075 | 0.073 | 0.024 | 0.026 | 0.109 | 0.073 | 0.151 | 0.144 |
| Ca (mg/Kg) | 391.061 | 64.259 | 378.849 | 67.352 | 3029.905 | 443.654 | 2949.836 | 411.081 | 162.373 | 44.091 | 28.795 | 21.741 | 1762.001 | 256.376 | 2737.676 | 432.201 |
| Fe (mg/Kg) | 134.434 | 38.869 | 125.534 | 17.290 | 878.324 | 431.754 | 497.458 | 90.930 | 56.928 | 23.704 | 8.988 | 5.369 | 507.201 | 246.463 | 462.811 | 96.589 |
| K (mg/Kg) | 3641.532 | 942.059 | 3915.887 | 410.891 | 5723.300 | 728.894 | 7011.389 | 1243.082 | 1508.523 | 542.608 | 276.378 | 163.726 | 3338.050 | 476.010 | 6504.783 | 1231.875 |
| Mg (mg/Kg) | 239.488 | 19.299 | 248.387 | 20.522 | 985.703 | 127.644 | 962.684 | 109.861 | 99.222 | 23.795 | 18.712 | 13.166 | 576.935 | 100.329 | 893.402 | 124.438 |
| Mn (mg/Kg) | 105.231 | 41.309 | 81.724 | 20.318 | 588.100 | 192.683 | 542.438 | 92.814 | 43.033 | 16.507 | 6.346 | 5.205 | 338.893 | 99.854 | 504.306 | 99.217 |
| Na (mg/Kg) | 81.020 | 19.635 | 96.123 | 38.560 | 128.991 | 39.336 | 111.982 | 35.863 | 33.499 | 11.787 | 7.805 | 7.501 | 74.756 | 21.756 | 102.864 | 29.094 |
| P (mg/Kg) | 430.589 | 106.178 | 469.159 | 114.547 | 1534.376 | 132.423 | 1830.399 | 165.331 | 181.267 | 73.250 | 33.645 | 21.651 | 899.388 | 141.086 | 1696.445 | 183.664 |
| Cd (mg/Kg) | 0.069 | 0.032 | 0.051 | 0.024 | 0.085 | 0.031 | 0.053 | 0.025 | 0.028 | 0.011 | 0.004 | 0.004 | 0.051 | 0.023 | 0.049 | 0.024 |
| Cr (mg/Kg) | 5.546 | 1.088 | 6.182 | 1.807 | 23.902 | 7.916 | 23.159 | 5.440 | 2.318 | 0.782 | 0.425 | 0.241 | 14.026 | 5.219 | 21.574 | 5.606 |
| Cu (mg/Kg) | 8.261 | 3.047 | 9.514 | 4.583 | 14.536 | 4.656 | 17.543 | 3.738 | 3.519 | 2.028 | 0.771 | 0.831 | 8.385 | 2.255 | 16.330 | 3.837 |
| Pb (mg/Kg) | 1.378 | 0.604 | 1.143 | 0.318 | 5.742 | 2.353 | 4.043 | 1.412 | 0.573 | 0.274 | 0.082 | 0.058 | 3.331 | 1.383 | 3.778 | 1.406 |
| Zn (mg/Kg) | 31.401 | 7.783 | 27.876 | 4.417 | 70.263 | 12.504 | 67.111 | 10.706 | 12.946 | 3.895 | 2.103 | 1.484 | 41.054 | 8.123 | 62.191 | 10.253 |
| Co2286 (mg/Kg) | 0.102 | 0.040 | 0.107 | 0.017 | 0.533 | 0.222 | 0.418 | 0.109 | 0.042 | 0.017 | 0.008 | 0.005 | 0.311 | 0.137 | 0.391 | 0.113 |
| Co2388 (mg/Kg) | 0.454 | 0.042 | 0.403 | 0.044 | 1.003 | 0.296 | 0.788 | 0.226 | 0.187 | 0.040 | 0.028 | 0.016 | 0.586 | 0.183 | 0.733 | 0.227 |
| Mo2020 (mg/Kg) | 0.118 | 0.056 | 0.165 | 0.055 | 0.258 | 0.061 | 0.241 | 0.077 | 0.047 | 0.021 | 0.013 | 0.012 | 0.150 | 0.036 | 0.222 | 0.065 |
| Mo2816 (mg/Kg) | 1.096 | 0.130 | 1.038 | 0.329 | 2.044 | 0.712 | 1.891 | 0.408 | 0.455 | 0.123 | 0.076 | 0.056 | 1.186 | 0.419 | 1.762 | 0.428 |
| Ti3234 (mg/Kg) | 4.945 | 1.309 | 4.416 | 0.858 | 30.419 | 16.758 | 24.334 | 10.017 | 2.051 | 0.772 | 0.303 | 0.158 | 17.669 | 10.427 | 22.801 | 9.857 |
| Ti3349 (mg/Kg) | 5.150 | 1.372 | 4.164 | 0.767 | 32.056 | 17.541 | 20.733 | 6.841 | 2.134 | 0.797 | 0.296 | 0.174 | 18.624 | 10.933 | 19.379 | 6.899 |
| Tannin (mmol/Kg) | 3.110 | 0.593 | 2.932 | 0.252 | 8.703 | 2.485 | 6.937 | 1.398 | 1.255 | 0.249 | 0.212 | 0.135 | 5.082 | 1.453 | 6.465 | 1.477 |
| Total phenol (mmol/Kg) | 6.961 | 1.122 | 6.000 | 0.623 | 18.920 | 3.087 | 14.670 | 2.266 | 2.843 | 0.634 | 0.446 | 0.309 | 11.049 | 1.996 | 13.609 | 2.273 |
